# [Retracted] MicroRNA-1236-3p/translationally controlled tumor protein (TPT1) axis participates in congenital hypothyroidism progression by regulating neuronal apoptosis

**DOI:** 10.3892/etm.2026.13142

**Published:** 2026-03-30

**Authors:** Tingting Meng, Shiman Shen, Cheng Li, Xuehua Liu

Exp Ther Med 19:459–466, 2020; DOI: 10.3892/etm.2019.8262

Following the publication of this paper, it was drawn to the Editor’s attention by a concerned reader that the western blot data shown in Fig. 2C were strikingly similar to data which had been submitted for publication to the same journal (*Experimental and Therapeutic Medicine*) a few months earlier in a paper written by different authors at different research institutes, which is to be retracted, albeit these very similar-looking data in that paper were shown in the horizontally reversed orientation. In addition, the presentation of flow cytometric assay data was in a style that matched that of a range of articles written by different authors at different research institutes, a few of which had already been submitted for publication prior to the receipt of this article at *Experimental and Therapeutic Medicine*, and some of which are destined to be retracted. Moreover, there appeared to be certain logical inconsistencies in the way in which the animal experiments in this paper were reported to have been conducted.

Owing to the fact that the contentious western blot data in the above article were found to be strikingly similar to data that had been submitted for publication elsewhere, the Editor of *Experimental and Therapeutic Medicine* has decided that this paper should be retracted from the Journal. The authors were asked for an explanation to account for these concerns, but the Editorial Office did not receive a reply. The Editor apologizes to the readership for any inconvenience caused.

